# Editorial: Privacy-Preserving Deep Heterogeneous View Perception for Data Learning

**DOI:** 10.3389/fnbot.2022.862535

**Published:** 2022-03-18

**Authors:** Peng Li

**Affiliations:** School of Software Technology, Dalian University of Technology, Dalian, China

**Keywords:** heterogenous data, deep learning, privacy-preserving neural computing, deep fuzzy perception, deep multi-view perception

Deep learning has promoted the development of cutting-edge robotic systems with the ability to automatically mine concepts from complex tasks in an open-ended manner. Many novel algorithms and efficient architectures of deep learning with trainable components have achieved remarkable performance in various domains such as machine learning and robotic devices, based on the unsupervised/supervised learning schemes.

Most of the current deep learning methods focus on a single-view perception of objects without fully considering the intrinsic characteristics of data, through which objects can be described by heterogeneous views. Those heterogeneous views contain complementary knowledge and information that can further improve representation learning of data. With development for easier access to heterogeneous view data promoted by wider deployments of edge-computing robotic devices, deep heterogeneous view perception distilling knowledge from various views is increasingly attracting more attention. At the same time, heterogeneous view data contains more private information than single view data. Mining large-scale heterogeneous view data inevitably raises the issue of privacy. With the emergence of deep heterogeneous view perception, privacies hidden in data are becoming more easily leaked. Thus, perception of deep heterogeneous view knowledge of data with preserving privacies is also becoming central to neural computing.

This Research Topic collects 8 high-quality articles reporting the latest applications of privacy-preserving deep heterogeneous learning. Below is a review of the articles published in this collection.

1. The paper by Chen and Yang focuses on the problem of robust feature extraction. The authors propose a novel multi-feature fusion method to merge information of heterogenous-view data for oil painting image feature extraction and recognition. This new method leverages the fusion of multiple heterogenous features with the help of Faster R-CNN. This method was evaluated on the benchmark and real data.
2. The paper by Chen, Jin et al. discusses the interpretability of deep representations. The authors introduce a deep matrix factorization method with non-negative constraints to learn deep part-based representations of interpretability for big data. This new method devises a deep supervisor network along with an interpretability loss. The superiority of the method was evaluated on two benchmark datasets.
3. The paper by Zhang et al. aims to improve the quality of low-light images. This paper proposed a heterogenous low-light image enhancement method based on the generative adversarial network. This new method utilizes the adversarial learning of distributions between low-light and normal-light images to enhance low-light image recognition. This method evaluated three benchmark datasets.
4. The paper by Yang et al. focuses on action recognition in complex environments. This paper proposes an improved residual dense neural network method to study the automatic recognition of dance action images. This method devises a new residual network, accompanied by the exponential linear element activation function, batch normalization, and dropout technology. This method was evaluated on the benchmark and real data.
5. The paper by Yan et al. focuses on the problem of data abnormalities in traditional multi-modal heterogeneous big data. The authors propose a multi-view K-means for heterogeneous big data. This method uses a BP neural network to predict the missing attribute values, complete the missing data, and denoise the abnormal data. This method was evaluated on the Iris data.
6. The paper by Liu H. et al. examines the benefits of deep heterogeneous view perception in teaching and education. The authors propose a 3-D multiscale residual dense network from heterogeneous view perception for analyzing student behavior recognition in class. This method was evaluated on the KTH, UCF-101, and the real scene data.
7. The paper by Liu D. et al. considers the interest recommendation from heterogeneous perspectives. The authors propose a privacy protection point of interest recommendation algorithm based on multi-exploring locality-sensitive hashing. The superiority of the method was evaluated on two benchmark datasets.
8. The paper by Chen, Zhang et al. explores challenges in new tasks of insufficient samples. The authors proposed a cross-modal method to realize context-awareness transfer in a few-shot image classification scene, which fully utilizes the information in heterogeneous data. The method was evaluated on two benchmark datasets.

## Author Contributions

The author confirms being the sole contributor of this work and has approved it for publication.

## Conflict of Interest

The author declares that the research was conducted in the absence of any commercial or financial relationships that could be construed as a potential conflict of interest.

## Publisher's Note

All claims expressed in this article are solely those of the authors and do not necessarily represent those of their affiliated organizations, or those of the publisher, the editors and the reviewers. Any product that may be evaluated in this article, or claim that may be made by its manufacturer, is not guaranteed or endorsed by the publisher.

